# ‘Science by consensus’ impedes scientific creativity and progress: An alternative to funding biomedical research

**DOI:** 10.12688/f1000research.124082.1

**Published:** 2022-08-19

**Authors:** Nejat Düzgüneş

**Affiliations:** 1 Department of Biomedical Sciences, University of the Pacific - San Francisco Campus, San Francisco, CA, 94103, USA

**Keywords:** Peer review; grant applications;NIH;NSF;granting agency

## Abstract

The very low success rates of grant applications to the National Institutes of Health (NIH) and the National Science Foundation (NSF) are highly detrimental to the progress of science and the careers of scientists. The peer review process that evaluates proposals has been claimed arbitrarily to be the best there is. This consensus system, however, has never been evaluated scientifically against an alternative. Here we delineate the 15 major problems with the peer review process, and challenge the Science Advisor to the President, and the leadership of NIH, NSF, and the U.S. Academy of Sciences to refute each of these criticisms. We call for the implementation of more equitable alternatives that will not constrain the progress of science. We propose a system that will fund 80,000 principal investigators, including young scientists, with just half the current NIH budget, three-fold more than the current number of grants, and that will forego the cumbersome, expensive, and counterproductive peer review stage. Further, we propose that the success of the two methods over 5–10 years be compared scientifically.

## Introduction

The success rate for National Institutes of Health (NIH) grants is currently 20% ( NIH Report, 2022). The funding rate at the National Science Foundation (NSF) was 26% in 2021 ( National Science Foundation, 2022). The Gates Foundation does not even release its grant success rate information. In 2009 and 2010, NIH received more than 20,000 applications for its Challenge Grants funded through the American Recovery and Reinvestment Act; the success rate was only 4% (NIH Report, 2011). The ‘successful’ projects are those that have been deemed by the consensus of peers to be worthwhile pursuing. Despite these very low percentages that afflict the careers of the great majority of scientists, the peer review system has been claimed to be the best system there is to allocate funding for biomedical research. This consensus system, however, has never been evaluated scientifically against an alternative ( Düzgüneş, 1999).

Perhaps the earliest challenge to this system at NIH was made by John McGowan (1992), who was at the time the Director of Extramural Research at the National Institute of Allergy and Infectious Diseases (NIAID). He revealed that proposals to investigate human immunodeficiency virus (HIV) infections of macrophages had been rejected by a study section because “the literature does not support the hypothesis that HIV can grow in macrophages” ( McGowan, 1992). And this is untrue! Regrettably, study sections have had too much power over what projects should proceed and which ones should be scrapped. As we have stated before, “such ‘science by consensus’ is unhealthy for the unfettered and productive pursuit of biomedical science” ( Düzgüneş, 1999).

We challenge the Science Advisor to the President, and the leadership of NIH, NSF, and the U.S. National Academy of Sciences to refute each of the following 15 major problems with the current NIH and NSF grant systems. If they cannot, however, and we believe they cannot, we ask these institutions to implement more equitable alternatives that will not constrain the progress of science.

### Problems with peer review

The NIH Peer Review document describes the mission of NIH to be seeking ‘fundamental knowledge about the nature and behavior of living systems and to apply that knowledge to enhance health, lengthen life, and reduce illness and disability.’ The document claims that the “NIH has a longstanding and time-tested system of peer review to identify the most promising biomedical research” ( NIH Peer Review, 2019). During the initial peer review, the scientific merit of a grant application is evaluated by the Scientific Review Group that comprises scientists with relevant expertise in the area. The second review is the responsibility of the National Advisory Councils or Boards that decide on funding a proposal as well as on research priorities. Despite the claims of NIH that this is a longstanding and time-tested review process, it has never been compared scientifically to an alternative system, with respect to scientific productivity and breakthroughs, new therapeutic modalities, patents and its psychological, personal and scientific impact on grant applicants who do not ‘succeed.’

Furthermore, NIH has to process over 80,000 applications a year, utilizing over 25,000 reviewers ( NIH Peer Review, 2019).

We have identified 15 major shortcomings and problems of peer review, which we delineate below.

*Some major breakthroughs in biomedical sciences have not been funded by NIH or NSF.* There have been several publicized cases of highly important research not being given grant support that have later gone on to be recognized as significant scientific discoveries. Nobel Prize winner Stan Prusiner was not able to obtain NIH funding for studying prions early on in his research. Craig Venter’s proposal to apply his whole-genome sequencing method to sequence a bacterial genome was not funded by NIH, and Nobel Prize winner Leon Cooper’s work on neural networks was not supported by either the NIH or NSF ( Bendiscioli, 2019). These examples should have been a history lesson for funding organizations like NIH and NSF ( Düzgüneş, 1998).

*Grant reviewers are competitors of applicants.* If they are truly ‘peers’, grant review panel members are very likely to be competitors of the grant applicant, even if not directly on the subject of the proposal. Thus, they will not be inclined to give the benefit of the doubt to an innovative research proposal that has not already been substantially carried out, particularly when they are struggling to procure funding themselves.

*Discoveries are made before grant awards.* The requirement for preliminary data in most grant applications indicates that a scientific discovery is expected to have already been made. Thus, the NIH and NSF may not be funding discoveries, but merely funding “mopping up operations,” in the words of Thomas Kuhn (1962), unless the preliminary data have been generated by a previous grant.

*Reviewer critiques may be inaccurate, but without the responsibility of making inaccurate statements.* Reviewers appear to have a mission to severely criticize applications to be able to weed them out, usually without the requirement to provide a published reference for any criticism. The reviewers are never accountable for their false statements or their scores ( Swift, 1996), even though they can derail scientific careers and the advancement of a field of science.

*Criticism never ends.* Grant applicants may re-apply after revising their proposal to respond to the written critique of the review panel. However, the panel may have new members at this later time and may then have entirely new criticisms. In essence, if the review panel does not want to fund an application it will not fund the application, revealing the whims of the individual reviewers.

Early career reviewers trained in a narrow area of science often think that valid science is what they are trained in. Thus, they prevent the progress of science that may otherwise produce significant insights or therapeutic approaches to treat diseases. This problem was emphasized by Costello (2010): “… the new generation of grant reviewers judge grant proposals through the myopic lenses of their specialties …. Important ideas and proposals that lie outside the current interest in molecular biology are unlikely to get a credible and knowledgeable review …”

*Nonscientific, unpublished review criteria.* Reviewers tend to use nonscientific criteria when making funding decisions. These include: (i) ‘probability of success’ which would favor projects proposing only incremental advances and no risk-taking; (ii) ‘level of enthusiasm’ which is highly subjective and depends on the reviewer’s mood at the time; and (iii) ‘grantsmanship’ which is essentially rendering grant-writing a game, expecting particular approaches to the project. Implying the nonscientific nature of the evaluation process, the study by Pier *et al.,* (2018) has shown that there is very little agreement between reviewers evaluating the same NIH grant applications.

*Translational projects may require long-term funding.* Projects that need additional time and experimentation to translate basic findings and initial discoveries into therapeutics or diagnostics may be considered by reviewers not to be innovative, thereby precluding the rapid development of a product that could diagnose or treat diseases. An example of this problem with NIH peer review is our inability to obtain grant funding since the mid-2000s for our research to develop gene therapy for oral cancer based on our initial discoveries ( Neves *et al.,* 2009), despite many applications.

*Robbing Peter to pay Paul.* Principal investigators may need to channel the funds of an existing grant to produce preliminary data for a new application in a new research area, instead of performing the funded experiments. Thus, experiments described in detail in applications may never be carried out and may essentially have been written only to convince reviewers to fund the grant application. In our view, this practice is unethical. It also demonstrates the absurdity of requiring preliminary data.

*Precious scientist time is wasted on grant applications.* Investigators spend a large proportion of their time on grant applications, which necessarily takes them away from their currently funded projects, if they are indeed grant recipients. This is not only counter-productive but may also be time paid by salary support from the granting agency, time that should have been spent on the funded project. A study by Kulage *et al.* (2015) calculated the cost of preparing a grant application. Principal investigators in this particular field spent between 70 to 162 hours per grant, and research administrators spent 34 to 66 hours, at a cost of USD $4,784 to $13,512. They estimated that, because funding rates are in the range 5–15%, a grant that is eventually funded would cost $72,460–$270,240. They concluded that “less costly and more efficient models of research funding are needed for the sustainability of the nursing profession” ( Kulage *et al.,* 2015). Scientists who have spent years in training and in research should be spending their time on scientific research, not on bureaucracy.

*Describing experiments to be performed in five years is unrealistic.* The elaborate description of experiments that will be performed three or five years in the future in a grant application contradicts the true nature of scientific research. Thus, for reviewers to expect meticulous descriptions, as if this is how science advances, goes against the true nature of science. Science is driven by the insights of scientists and new discoveries, and often requires immediate changes in approach or direction.

*Waiting for grant funding hinders scientific progress.* Many fields advance rapidly while investigators are waiting for their grant applications to be evaluated and funded. If the investigator is not funded independently, the project barely moves forward. We cannot afford science to progress at this slow and saltatory rate with the uncertainty in grant funding.

*The human and material cost of NIH peer review.* The administration of approximately 80,000 applications and 25,000 reviewers per year ( NIH Peer Review, 2019) costs NIH and the research community both money and time that could have been used for actual research. For reviewers, evaluating grant applications is a chore performed for the sake of recognition and prestige, and perhaps to increase their own chances of obtaining funding. This can result in the compromise of objectivity by the reviewers, and even resentment, because of the inordinate amount of time required to complete a review. NIH officials conducting sessions at scientific meetings on how to write grants admit that reviewers may not be able to spend quality time on reviewing applications. Of course, this is never admitted in print, since peer review is supposed to be unquestionably the ideal system for funding science.

*NIH scientists do not compete for grant funding.* Although NIH provides extramural funds following grueling peer review of grant applications, its own scientists do not have to compete for this type of funding. Thus, NIH itself appears to have recognized the extreme drawbacks of the peer review system, enabling its intramural community to undertake long-term projects with stable funding and large laboratory groups. If peer review is such an indispensable system for funding science, why does NIH not implement this system for its own scientists? Why is the extramural scientific community considered second-rate citizens who must clamor for funding all their lives?

*Review panel scores do not predict success.* An analysis of 102,740 funded grants has shown that percentile scores generated by NIH review panels for the applications are poor predictors of publication and citation productivity ( Fang *et al.,* 2016). Thus, the meticulous scoring process is essentially useless. Arturo Casadevall of Johns Hopkins University and the senior author of this study is quoted as saying “A negative word at the table can often swing the debate. And this is how we allocate research funding in this country” ( Johns Hopkins Bloomberg School of Public Health, 2016).

### Alternatives to peer review

We have previously proposed simple alternatives to the current peer review system ( Düzgüneş, 1999, 2007). This new system would provide continuous and stable funding for 10-year periods to scientists with a track record of solid publications ( Düzgüneş, 1999) and to young scientists starting their first independent positions in a university or a research institute ( Düzgüneş, 2007). Scientists opting for this mode of funding would merely submit a letter of intent with a one-page broad outline of their research direction. They could be chosen based on criteria including publications, citations and potential impact of their research field, by an *international* group of both established and young scientists *who are not in a position to receive funding from NIH or NSF* and are thus not competitors. About half of the NIH extramural funds could still be allocated to the current system of review, especially for projects requiring much larger budgets than what we are proposing below.

Under this new system, NIH grants to established scientists would be limited to $400,000 per year, and they would be phased in over several years, up to possibly 40,000 grantees. With indirect costs limited to 30%, these grants would cost $20.8 billion per year. Grants to young investigators would be set at $150,000 per year, with the same indirect cost rate. 40,000 such grants would cost NIH $7.8 billion. Thus, at a total cost of $28.6 billion the *NIH could fund 80,000 such grants*, with minuscule expenses for scientific review. Since the sum is only slightly more than half of the current NIH budget of $52 billion, the rest of the NIH budget could be allocated to about *half* (about 13,000) the current number of grants. Since this system will be phased in, and the NIH budget is likely to increase within the next five years, there will be no undue burden on the traditional grants and intramural funding. This system will result in the funding of 93,000 principal investigators, instead of the current approximately 26,000.

It is instructive to note the findings of Azoulay *et al.* (2011) in comparing Howard Hughes Medical Institute awardees and NIH grant recipients. They reported that “selection into the HHMI investigator program—which rewards long-term success, encourages intellectual experimentation, and provides rich feedback to its appointees—leads to higher levels of breakthrough innovation, compared with NIH funding—which is characterized by short grant cycles, predefined deliverables, and unforgiving renewal policies. Moreover, the magnitudes of these effects are quite large.”

Vaesen and Katzav (2017) analyzed the proposal to “distribute available funds equally among all qualified researchers, with no interference from peer review.” Their analysis indicated that “researchers could, on average, maintain current PhD student and Postdoc employment levels, and still have at their disposal a moderate (the U.K.) to considerable (the Netherlands, U.S.) budget for travel and equipment.” Our proposal combines this equitable distribution of funds with the option for scientists undertaking very expensive projects to apply for the remaining highly competitive funds.

### Evaluating the scientific success of grants obtained via peer review and the alternative system proposed here

The paradigm shift we are proposing does not end here. The scientific productivity of scientists in these two categories over a five-year and 10-year period will be analyzed, in terms of citations, significant discoveries, and development of therapeutics, per dollar amount spent. As we have indicated previously ( Düzgüneş, 1999), “The United States has expended enormous capital in the training of its scientists. The scientific potential of the more than 80 percent of biomedical scientists who are unable to procure grants is too precious a resource to waste.”

## Conclusions

While contemplating writing this section, we came across an e-mail sent to potential NIH grant applicants and a separate website aimed at academics and including advice on grant applications as part of an industry aimed at grant applicants for ‘winning’ reviews. The e-mail advertised that their program enabled the participants to ‘successfully write for reviewers’. If an applicant is writing to impress a particular reviewer, the detailed norms, supposed objectivity, and scoring system of NIH peer review becomes questionable. Another website gave the advice to involve the reviewers’ ‘reptilian brain’ and went on to say that the written review of a grant application may come from the rational, cerebral layer of the brain, but the decision on whether the grant is awarded or not actually comes from the most instinctual layer. What has become of the best method to review grant applications?

With all the problems of peer review of grant applications, we ask the Science Advisor to the President, and the leadership of NIH, NSF, and the U.S. National Academy of Sciences to implement more equitable alternatives that will not constrain the progress of science. A staring point is the very simple and highly cost-effective alternative we have proposed here.

## Data availability

No data are associated with this article.
