# ‘Science by consensus’ impedes scientific creativity and progress: A simple alternative to funding biomedical research

**DOI:** 10.12688/f1000research.124082.3

**Published:** 2024-02-21

**Authors:** Nejat Düzgüneş

**Affiliations:** 1 Department of Biomedical Sciences, University of the Pacific - San Francisco Campus, San Francisco, CA, 94103, USA

**Keywords:** Peer review; grant applications;NIH;NSF;granting agency

## Abstract

The very low success rates of grant applications to the National Institutes of Health (NIH) and the National Science Foundation (NSF) are highly detrimental to the progress of science and the careers of scientists. The peer review process that evaluates proposals has been claimed arbitrarily to be the best there is. This consensus system, however, has never been evaluated scientifically against an alternative. Here we delineate the 15 major problems with the peer review process. We challenge the Science Advisor to the President, and the leadership of NIH, NSF, the U.S. National Academy of Sciences and other funding agencies throughout the world to refute each of these criticisms. We call for the implementation of more equitable alternatives that will not constrain the progress of science. We propose a system that will fund at least 80,000 principal investigators, including young scientists, with about half the current NIH budget, seven-times as many as the current number of NIH “research project grants,” and that will forego the cumbersome, expensive, and counterproductive “peer” review stage. Further, we propose that the success of the two systems over 5–10 years be compared scientifically.

## Introduction

The success rate for National Institutes of Health (NIH) grants is currently 20% ( NIH Report, 2022), and the “payline” for research grant applications by experienced principal investigators is 11% ( NIAID Funding News, 2023). The funding rate at the National Science Foundation (NSF) was 26% in 2021 ( National Science Foundation, 2022). The Gates Foundation does not even release its grant success rate information; it may be between 1 to 2%. In 2009 and 2010, NIH received more than 20,000 applications for its Challenge Grants funded through the American Recovery and Reinvestment Act; the success rate was only 4% (NIH Report, 2011). The “successful” projects are those that have been deemed by the consensus of “peers” to be worthwhile pursuing; hence, our designation for the way science appears to be carried out: “science by consensus.” Even the designation, “peer review” is suspect, because review committees comprise many scientists who do not adequately know the field of the grant application.

Despite these very low success rates and paylines that afflict the careers of the great majority of scientists, the peer review system has been claimed to be the best system there is to allocate funding for biomedical research (see, for example, Racker, 1979). This consensus system, however, has never been evaluated scientifically against an alternative ( Düzgüneş, 1999). Thus, the reputation of the current system is not based on systematic comparisons against other unbiased and more rational systems.

Perhaps the earliest challenge to this system at NIH was made by John McGowan (1992), who was at the time the Director of Extramural Research at the National Institute of Allergy and Infectious Diseases (NIAID). He revealed that proposals to investigate human immunodeficiency virus (HIV) infections of macrophages had been rejected by a study section because “the literature does not support the hypothesis that HIV can grow in macrophages” ( McGowan, 1992). And this is untrue! Regrettably, study sections have had too much power over what projects should proceed and which ones should be scrapped. As we have stated before, “such ‘science by consensus’ is unhealthy for the unfettered and productive pursuit of biomedical science” ( Düzgüneş, 1999).

We challenge the Science Advisor to the President, and the leadership of NIH, NSF, and the U.S. National Academy of Sciences, and funding agencies throughout the world to refute each of the following 15 major problems with the current NIH and NSF grant systems. If they cannot, however, and we believe they cannot, we ask these institutions to implement more equitable alternatives that will not constrain the progress of science.

### Problems with peer review

The NIH Peer Review document describes the mission of NIH to be seeking “fundamental knowledge about the nature and behavior of living systems and to apply that knowledge to enhance health, lengthen life, and reduce illness and disability.” The document claims that the “NIH has a longstanding and time-tested system of peer review to identify the most promising biomedical research” ( NIH Peer Review, 2019). During the initial peer review, the scientific merit of a grant application is evaluated by the Scientific Review Group that comprises scientists with relevant expertise in the area. The second review is the responsibility of the National Advisory Councils or Boards that decide on funding a proposal as well as on research priorities. Despite the claims of NIH that this is a longstanding and time-tested review process, it has never been compared scientifically to an alternative system, with respect to scientific productivity and breakthroughs, new therapeutic modalities, patents and its psychological, personal and scientific impact on grant applicants who do not “succeed.”

Furthermore, NIH has to process over 80,000 applications a year, utilizing over 25,000 reviewers ( NIH Peer Review, 2019), an extremely wasteful system, possibly costing $1 billion/year, including the efforts of grant applicants and their post-doctoral fellows, graduate students and administrative assistants ( Pagano, 2006).

Here we expound the major shortcomings and problems of peer review as it applies to the evaluation of grant applications.

*Some major breakthroughs in biomedical sciences have not been funded by NIH or NSF.* There have been several publicized cases of highly important research not being given grant support that have later gone on to be recognized as significant scientific discoveries. Nobel Prize winner Stan Prusiner was not able to obtain NIH funding for studying prions early on in his research ( Düzgüneş, 1998). Craig Venter’s proposal to apply his whole-genome sequencing method to sequence a bacterial genome was not funded by NIH, and Nobel Prize winner Leon Cooper’s work on neural networks was not supported by either the NIH or NSF ( Bendiscioli, 2019). The most recent example is the work of Katalin Karikó, the winner of the Nobel Prize in Physiology or Medicine in 2023, who could not obtain funding for her groundbreaking work while at the University of Pennsylvania ( Mueller & Kolata, 2023). These examples should have been a history lesson for funding organizations like NIH and NSF, which we pointed out 25 years ago ( Düzgüneş, 1998)!

*Grant reviewers are competitors of applicants.* If they are truly “peers”, grant review panel members are very likely to be competitors of the grant applicant, even if not directly on the subject of the proposal. Thus, they will not be inclined to give the benefit of the doubt to an innovative research proposal that has not already been substantially carried out, particularly when they are struggling to procure funding themselves.

*Discoveries are made before grant awards.* The requirement and expectations for preliminary data in most grant applications indicates that a scientific discovery is expected to have already been made. Thus, the NIH and NSF may not be funding discoveries, but merely funding “mopping up operations,” in the words of Thomas Kuhn (1962), unless the preliminary data have been generated by a previous grant, which may have been on an entirely different subject.

*Reviewer critiques may be inaccurate, but without the responsibility and accountability of making inaccurate statements.* Reviewers appear to have a mission to severely criticize applications to be able to weed them out, usually without the requirement to provide a published reference for any criticism. The reviewers are never accountable for their false statements or their scores ( Swift, 1996), even though they can derail scientific careers and the advancement of a field of science.

*Criticism never ends.* Grant applicants may re-apply after revising their proposal to respond to the written critique of the review panel. However, the panel may have new members at this later time and may then have entirely new criticisms. In essence, if the review panel does not want to fund an application, it will find ways not to fund the application, revealing the whims of the individual reviewers.

Early career reviewers trained in a narrow area of science often think that valid science is what they are trained in. Thus, they may prevent the progress of science that may otherwise produce significant insights or therapeutic approaches to treat diseases. This problem was emphasized by Costello (2010): “… the new generation of grant reviewers judge grant proposals through the myopic lenses of their specialties …. Important ideas and proposals that lie outside the current interest in molecular biology are unlikely to get a credible and knowledgeable review …”

*Nonscientific, unpublished review criteria.* Reviewers tend to use nonscientific criteria when making funding decisions. These include: (i) “probability of success” which would favor projects proposing only incremental advances and no risk-taking; (ii) “level of enthusiasm” which is highly subjective and depends on the reviewer’s mood at the time; and (iii) “grantsmanship” which is essentially rendering grant-writing a game, expecting particular approaches to the project. Implying the nonscientific nature of the evaluation process, the study by Pier *et al.,* (2018) has shown that there is very little agreement between reviewers evaluating the same NIH grant applications.

*Translational projects may require long-term funding.* Projects that need additional time and experimentation to translate basic findings and initial discoveries into therapeutics or diagnostics may be considered by reviewers not to be innovative, thereby precluding the rapid development of a product that could diagnose or treat diseases. An example of this problem with NIH peer review is our inability to obtain grant funding since the mid-2000s for our research to develop gene therapy for oral cancer based on our initial discoveries ( Neves *et al.,* 2009), despite many applications.

*Robbing Peter to pay Paul.* Principal investigators may need to channel the funds of an existing grant to produce preliminary data for a new application in a new research area, instead of performing the funded experiments. Thus, experiments described in detail in applications may never be carried out and may essentially have been written only to convince reviewers to fund the grant application. In our view, this practice is unethical. It also demonstrates the absurdity of requiring preliminary data.

*Precious scientist time is wasted on grant applications.* Investigators spend a large proportion of their time on grant applications, which necessarily takes them away from their currently funded projects, if they are indeed grant recipients. This is not only counter-productive but may also be time paid by salary support from the granting agency, time that should have been spent on the funded project. A study by Kulage *et al.* (2015) calculated the cost of preparing a grant application. Principal investigators in this particular field spent between 70 to 162 hours per grant, and research administrators spent 34 to 66 hours, at a cost of USD $4,784 to $13,512. They estimated that, because funding rates are in the range 5–15%, a grant that is eventually funded would cost $72,460–$270,240. They concluded that “less costly and more efficient models of research funding are needed for the sustainability of the nursing profession” ( Kulage *et al.,* 2015). Scientists who have spent years in training and in research should be spending their time on scientific research and not on bureaucracy.

*Describing experiments to be performed in five years is unrealistic.* The elaborate description of experiments that will be performed three or five years in the future in a grant application contradicts the true nature of scientific research. “Indeed, if the scientific enterprise were predictable, science would be banal, perhaps even boring” ( Pagano, 2006). Thus, for reviewers to expect meticulous descriptions, as if this is how science advances, goes against the true nature of science. Science is driven by the insights of scientists and new discoveries, and often requires immediate changes in approach or direction.

*Waiting for grant funding hinders scientific progress.* Many fields advance rapidly while investigators are waiting for their grant applications to be evaluated and funded. If the investigator is not funded independently, the project barely moves forward. With the uncertainty in grant funding, we cannot afford science to progress at this slow and saltatory rate.

*The human and material cost of NIH peer review.* The administration of approximately 80,000 applications and 25,000 reviewers per year ( NIH Peer Review, 2019) costs NIH and the research community both money and time that could have been used for actual research. Pagano (2006) has estimated that the cost of the preparation of grant applications in terms of the salaries of the applicants, their post-doctoral fellows, students and administrative assistants, and the time spent by all the reviewers could be more than $1 billion/year. For reviewers, evaluating grant applications is a chore performed for the sake of recognition and prestige, and perhaps to increase their own chances of obtaining funding. This can result in the compromise of objectivity by the reviewers, and even resentment, because of the inordinate amount of time required to complete a review. NIH officials conducting sessions at scientific meetings on how to write grant applications admit that reviewers may not be able to spend quality time on reviewing applications. Of course, this is never admitted in print, since peer review is supposed to be unquestionably the ideal system for funding science.

*NIH scientists do not compete for grant funding.* Although NIH provides extramural funds following grueling peer review of grant applications, its own scientists do not have to compete for this type of funding. Thus, NIH itself appears to have recognized the extreme drawbacks of the peer review system, enabling its intramural community to undertake long-term projects with stable funding and large laboratory groups. If peer review is such an indispensable system for funding science, why does NIH not implement this system for its own scientists? Why is the extramural scientific community considered second-rate citizens who must clamor for funding all their lives?

*Review panel scores do not predict success.* An analysis of 102,740 funded grants has shown that percentile scores generated by NIH review panels for the applications are poor predictors of publication and citation productivity ( Fang *et al.,* 2016). Thus, the meticulous scoring process is essentially useless. Arturo Casadevall of Johns Hopkins University and the senior author of this study is quoted as saying “A negative word at the table can often swing the debate. And this is how we allocate research funding in this country” ( Johns Hopkins Bloomberg School of Public Health, 2016).

### “Interventions” to peer review

An extensive study, published after the original version of the current paper was submitted, listed 38 “interventions” to peer review that would remedy its shortcomings ( Kolarz *et al.*, 2023). These interventions included limiting the number of applications from an institution, “dragon’s den style pitch,” interviews, moderation panels, applicants assessing proposals from other applicants, wildcard by each panel member to ensure funding of a particular project, sequential application of funding criteria, use of quotas, calling out unconscious biases, training “good reviewers,” and open review and rebuttal. In this study, proponents of peer review appear to be grasping at straws to save peer review.

The frustration with the shortcomings of peer review has led some funding agencies, including the Health Research Council of New Zealand and the Volkswagen Foundation in Germany to revert to random selection of grantees by lottery ( Avin, 2019). Schaubroeck (2022) has argued for a mixture of competitive and non-competitive funding. The system proposed in the next section is in line with this reasoning.

### A simple and rational alternative to peer review

We previously proposed a simple alternative to the current peer review system ( Düzgüneş, 1999, 2007). This new system would provide continuous and stable funding for 10-year periods to scientists with a track record of solid publications ( Düzgüneş, 1999) and to young scientists starting their first independent positions in a university or a research institute ( Düzgüneş, 2007). Scientists opting for this mode of funding would merely submit a letter of intent with a one-page broad outline of their research direction. They could be chosen based on criteria including publications, citations and potential impact of their research field, by an *international* group of both established and young scientists *who are not in a position to receive funding from NIH or NSF* and are thus not competitors, would establish objective criteria for funding. These scientists would not act as peer reviewers of any grant applicant or application. They would merely set the objective criteria by which scientists applying for the new system are ranked, without knowing anything about the scientist, their field of research or the general topic of the research the scientist wishes to pursue. The criteria may include the number of citations and publications, the *d*-index ( Di Caro *et al.*, 2012), the *h*-index ( Hirsch, 2005), and other factors the panel may consider to be useful. If such ranking is too rigorous and eliminates too many of the applicants (which could be calculated almost instantaneously), the panel would relax the criteria to include more of the applicants.

Under this new system, NIH grants to scientists with the appropriate ranking would be limited to $400,000 per year of direct costs. These grants would be phased in over several years, up to 40,000 grantees. With indirect costs limited to 30%, all of these grants would cost $20.8 billion per year. Investigators who opt for this system would agree not to apply for the competitivie grants, so that they would not have an unfair advantage, although they could apply for shared instrument, center, or training grants. If they opt to obtain increased funding through the competitive system, they would have to forego their alternative grant (except for the last 1 year) and struggle like the rest of their competitors!

Grants to young investigators would be set at $150,000 per year, with the same indirect cost rate. Forty thousand such grants would cost NIH $7.8 billion. Thus, at a total cost of $28.6 billion the *NIH could fund 80,000 such grants*, with minuscule expenses for scientific review.

Since individual applicants will not be identified during the establishment of the objective criteria, it will be impossible for there to be any discrimination towards the applicant on the basis of gender or minority status.

NIH currently funds 11,311 research project grants (“RPG”) at a cost of $24.4 billion ( https://nexus.od.nih.gov/all/2023/03/01/fy-2022-by-the-numbers-extramural-grant-investments-in-research/ (accessed 28 October 2023). Of these, only 7,816 are R01-equivalent grants. The system we are proposing would thus fund about *7 times as many research grants* as NIH currently does.

To accommodate the budget of the 80,000 grants, the number of competitive grants would be initially cut in half, to about 5,655 RPGs, at a cost of $12.2 billion. Since the proposed system will be phased in, and the NIH budget is likely to increase within the next few years, we anticipate that there will be no undue burden on the traditional grants and intramural funding at NIH, as the proposed system is implemented. In fact, competition for funds in the traditional system would be drastically reduced because there would be 7-fold fewer grant applicants who would have opted for the new system.

### Possible unintended consequences of the alternative system

Would there be unintended consequences of implementing the proposed alternative? It is theoretically possible, but extremely unlikely, that a scientist’s track record is exaggerated by the availalble data, resulting in a grant award, and that they do not make any significant scientific progress during the 10 years of funding. Although this is highly unlikely, the loss of scientific progress in these circumstances should be compared to the loss of productivity by a laboratory that cannot obtain funding or cannot renew a grant, resulting in the departure from the laboratory of the trained personnel, or closing of the laboratory altogether.

We can also envision a scientist who opted for the novel funding mechanism, but did not make the cutoff of scientific measures used to establish the track record. First, there could be an appeal for a review of the scientist’s track record. Even if this were to fail, the scientist still has the option to apply for the competitive grants. In fact, this is essentially the situation with the current peer review system, where about 80–90% of biomedical scientists find themselves. The frequency of instances of such unintended consequences, could be quantified by the granting agency. If these potentially very few instances are considered to be inefficiencies of the system we are proposing, we are inclined to ask if there is *any* efficiency in the current peer review system! We contend that, since the ranking or evaluation system is without reviewer bias (there being no reviewers), it is orders of magnitude more reliable than the “science by consensus” system.

### Other alternatives to the peer review system

It is instructive to note the findings of Azoulay *et al.* (2011) in comparing Howard Hughes Medical Institute awardees and NIH grant recipients. They reported that “selection into the HHMI investigator program—which rewards long-term success, encourages intellectual experimentation, and provides rich feedback to its appointees—leads to higher levels of breakthrough innovation, compared with NIH funding—which is characterized by short grant cycles, predefined deliverables, and unforgiving renewal policies. Moreover, the magnitudes of these effects are quite large.” Scientists chosen for this program have a track record of scientific productivity, similar to our proposal, which, however, will have a broader base of scientists.

Vaesen and Katzav (2017) analyzed the proposal to “distribute available funds equally among all qualified researchers, with no interference from peer review.” Their analysis indicated that “researchers could, on average, maintain current PhD student and Postdoc employment levels, and still have at their disposal a moderate (the U.K.) to considerable (the Netherlands, U.S.) budget for travel and equipment.” Our proposal combines this equitable distribution of funds with the option for scientists undertaking very expensive projects to apply for the remaining highly competitive funds.

### Evaluating the scientific success of grants obtained via peer review and the alternative system proposed here

The paradigm shift we are proposing does not end here. The scientific productivity of scientists in these two categories over a 5-year and 10-year period will be analyzed, in terms of publications, citations, significant discoveries, patents, the *d*-index and *h*-index of the grant recipients, and the development of therapeutics, per dollar amount spent. These criteria would be weighted by the same or similar international panel that established the initial cutoff criteria for grantees in the new system. This evaluation would then prove or disprove the long-held assumption that peer review is the best system to award NIH or NSF grants.

If the current peer review system is shown not to be superior to the alternative we are proposing here, would it be abandoned? We would hope so. What, then, would become of the projects that necessicate larger budgets? We suggest some possibilities: Several investigators in the alternative system would pool their resources to tackle such projects. Centers focusing on a particular health condition that require very large budgets, such as autism and Alzheimer’s disease centers, would be funded separately, possibly from the budget of the competitive grants. Investigators funded by this mechanisms, however, would not be eligible to be included in the alternative system we are proposing.

As we have indicated previously ( Düzgüneş, 1999), “The United States has expended enormous capital in the training of its scientists. The scientific potential of the more than 80 percent of biomedical scientists who are unable to procure grants is too precious a resource to waste.”

## Conclusions

While contemplating writing this section, we came across an e-mail sent to potential NIH grant applicants and a separate website aimed at academics and including advice on grant applications as part of an industry aimed at grant applicants for “winning” reviews. The e-mail advertised that their program enabled the participants to “successfully write for reviewers”. If an applicant is writing to impress a particular reviewer, the detailed norms, supposed objectivity, and scoring system of NIH peer review becomes questionable. Another website gave the advice to involve the reviewers’ “reptilian brain” and went on to say that the written review of a grant application may come from the rational, cerebral layer of the brain, but the decision on whether the grant is awarded or not actually comes from the most instinctual layer. What has become of the best method to review grant applications?

Considering all the problems of peer review of grant applications, we ask the Science Advisor to the President, and the leadership of NIH, NSF, the U.S. National Academy of Sciences and other funding agencies throughout the world to implement more equitable alternatives that will not constrain the progress of science. A staring point is the very simple and highly cost-effective alternative we have proposed here.

## Data availability

No data are associated with this article.
