# Supplementary material for: ‘Science by consensus’ impedes scientific creativity and progress: A simple alternative to funding biomedical research
Source: F1000Res. 2024 Feb 21;11:961. Originally published 2022 Aug 19. [Version 3] doi: 10.12688/f1000research.124082.3 (PMC11126901; doi:10.12688/f1000research.124082.3)
Supplement: Peer review history

# Reviewer report: Reviewer response for version 3

**DOI:** 10.5256/f1000research.162827.r248868

**Published:** 2024-05-25

**Authors:** Jamie Shaw

**Affiliations:** 1 Leibniz Universität Hannover, Toronto, Canada

In this paper, Professor Düzgüneş offers a fairly thorough review of the criticisms against peer review in science funding policy and challenges administrators at various institutions to address these criticisms or else change directions. Professor Düzgüneş further offers a rough-and-ready proposal for an alternative funding scheme that leads to a more egalitarian funding distribution.

 I too am of the opinion that peer review is in a great deal of peril. The more it is used, the more we risk suffering from its problems and so the urgency of Professor Düzgüneş’ challenge is entirely warranted. A response is also necessary, so that the NIH or NSF (or whoever) can contribute to the transparency of their decision making. I am, moreover, entirely sympathetic to more direct funding practices in the ballpark of those suggested by Professor Düzgüneş.

 Before providing some more critical remarks on this important paper, I’d like to highlight that I do not have Professor Fang’s expertise with regards to the necessary funding levels for individual labs and withhold judgment about whether the dollar amounts could be made to work. This is an important objection that must be answered satisfactorily. In any case, such a budgetary analysis would require some contextual focus since expenses in theoretical projects are quite different from those in experimental contexts (and even within experimentalists, there are those starting a new lab and those who are able to borrow/use existing infrastructure). This provides wiggle room for a more nuanced distribution recommendation but, again, I leave this discussion open to others who know more than me on this subject. My only point here is that this debate will only be substantive focused on more specific institutional policies.

 I am in agreement with Professor Fang’s remark about the presentation of the criticisms of peer review. The reason is that some of these objections, when isolated, are not genuine objections. For example, regarding the section under the heading “Some major breakthroughs in biomedical sciences have not been funded by NIH or NSF.” This point on its own, while rhetorically powerful, is not particularly strong. Given the sheer number of applications, the NIH is bound to miss some breakthroughs. The real question is whether these examples are symptomatic of deeper, systematic patterns. They are, and this point is raised latter, but this underlying problem is the objection – not the mere fact that they missed out on breakthroughs. I suggest merging these objections, rather than separating them. Similarly, “Robbing Peter to pay Paul” seems more like a corollary to “Discoveries are made before grant awards” than a separate objection.

 In other cases, the section header seems misleading. “Criticism never ends”, for example. The real worry here is that criticisms (also praise?) waver drastically amongst reviewers. The best up-to-date meta-study on studies on this front that I am aware of is Guthrie S et. al, 2017 (ref [5]). In other cases, the objections are most likely legitimate but lack citations (e.g., “Grant reviewers are competitors of applicants”, “Discoveries are made before grant awards”). While these points are often discussed informally, analytic treatment of them that has been published should be incorporated here. Finally, the objection that “Translational projects may require long-term funding” at least needs to acknowledge that the NIH and NSF (and many other institutions) have dedicated special attention to translational research that can be extremely sizeable. The criticism here seems also to not concern peer review, more over, but the amount of funds available for translational research. The same is true for “Describing experiments to be performed in five years is unrealistic” which, if true, is not a problem of peer review *per se *but a problem of what is expected to be reviewed.

 Finally, it seems like a missed opportunity in the section “Nonscientific, unpublished review criteria” to not spend some time discussing the myriads of biases that discriminate on the basis of gender, age, ethnicity, etc. This is a massive objection against peer review and should be noted with some detail here.

 With regards to the positive proposal, I have strong reservations about the proposed criteria for assessment focused (seemingly exclusively) on publications and citation counts. This would likely lead to inadvertent discrimination. Many scientists have CV gaps that aren’t a result of anything problematic (e.g., they had children, had to take care of their families, spent time doing public engagement instead of publishing, etc.). Not only should publications not be treated as a *sine qua non *of science and be used as a sole (or even primary basis) of funding, but ‘productivity’ measured in this way will marginalise already marginalized scientists who can be perfectly capable and talented members of the scientific community (see ref [3]; ref [2] ; ref [9] for starters). This will surely decrease the diversity of the scientific community as well which carries strong implications for innovation in science (Ginther DK. et. al, 2018 ref [4]) found that funding disparities amongst black and white applicants at the NIH were mostly caused by productivity gaps. Moreover, in the biomedical sciences in particular, there are worries that focus on productivity leads to more research funds going towards scientists with connections to for-profit companies which can bias their research (ref [6]; ref [7]). The same is true of citation counts, which are often not indicative of quality of work and stem from visibility issues or topic choices. Indeed, the authors motivation to improve innovation in science will likely be held back by promoting citation chasing, which often involves jumping on board with the latest trendy bandwagon. Moreover, and perhaps this is what Dr. Recio-Saucedo meant by “changing research culture”, we are increasingly coming to recognize the value of many activities that are not ‘productive’ in the sense of generating publications. Increased attention to pedagogy, public engagement, advisory work, and so on all become further disincentivized and marginalized if we make funding dependent on publications alone. There are also some research programs that do not demonstrate their value in terms of publications. For instance, research on public health in Indigenous communities on the west coast of Canada requires decades to properly integrate researchers into the community, understand local norms and needs, etc. and most of this work is not publishable (see Castleden et al. 2015). For these reasons, I strongly suggest moving either towards even more minimalistic criteria or at least incorporating a broader sense of what counts as being ‘productive.’

 Some smaller, but still important points include:

1. “possibly costing $1 billion/year, including the efforts of grant applicants and their post-doctoral fellows, graduate students and administrative assistants (Pagano, 2006).”
  1. This number is a guesstimate from 2006. It is probably much more expensive nowadays. A global study worth mentioning might be ref [1].
2. “probability of success” is a scientific criterion, although the author is correct to suggest that this metric favor conservative research.
3. “Review panel scores do not predict success”
  1. This is an oversimplification. In my meta-study, I saw some predictive success under the 5-year mark, but poor predictive success further into the future (ref [8]). That being said, I agree with Dr. Recio-Saucedo when they say that “Given the broad range of impacts that result from research, and the efforts on responsible use of metrics that are addressing known issues with research culture, the conclusion that a scoring system is not useful based on its ability to predict publication and citation productivity is to some extent reductive.” The studies cited in this meta-study use citation counts as proxies and I agree that this is too narrow of a criterion.
    1. Positive results were also found in ref [10]. These findings do not rely on citation counts.
4. “In this study, proponents of peer review appear to be grasping at straws to save peer review.”
  1. This is too dismissive of these efforts. Unless reasons can be given that these initiatives are not likely to make a major difference, this point is mere speculation. 
5. “science by consensus”
  1. This phrase might be misleading. The ‘consensus’ is amongst a very small group of ‘peers’ while some readers might associate ‘consensus’ with a broader community consensus. Perhaps ‘science by local consensus’ would be more appropriate.

I suggest a more recent reference than Racker (1979) for defending peer review as the best system there is.

 Peer review has been evaluating scientifically against alternatives quite a bit. Much of the papers cited in this paper do just this.

 I remain grateful for the opportunity to review and learn from this article, and believe is provides a valuable contribution to the growing chorus of discontent with peer review of grant applications. Urgent and potentially radical experimentation is necessary and I believe Professor Düzgüneş has taken admirable steps in this direction in this article as well as his earlier work. I very much hope that science funding institutions take notice.

Is the topic of the opinion article discussed accurately in the context of the current literature?

Yes

Are arguments sufficiently supported by evidence from the published literature?

Partly

Are all factual statements correct and adequately supported by citations?

Partly

Are the conclusions drawn balanced and justified on the basis of the presented arguments?

Partly

Reviewer Expertise:

I am a philosopher of science, engaged in multi and interdisciplinary research on peer review in science funding policy.

I confirm that I have read this submission and believe that I have an appropriate level of expertise to confirm that it is of an acceptable scientific standard, however I have significant reservations, as outlined above.

## References

[1] : A billion-dollar donation: estimating the cost of researchers' time spent on peer review. *Res Integr Peer Rev* .2021;6(1) : 10.1186/s41073-021-00118-2 14 10.1186/s41073-021-00118-2 34776003 PMC8591820

[2] : Unravelling the gender productivity gap in science: a meta-analytical review. *R Soc Open Sci* .2019;6(6) : 10.1098/rsos.181566 181566 10.1098/rsos.181566 31312468 PMC6599789

[3] : Motherhood and the Gender Productivity Gap. *SSRN Electronic Journal* .2018; 10.2139/ssrn.3198356 10.2139/ssrn.3198356

[4] : What do we know about grant peer review in the health sciences?. *F1000Res* .2017;6: 10.12688/f1000research.11917.2 1335 10.12688/f1000research.11917.2 29707193 PMC5883382

[5] : Publications as predictors of racial and ethnic differences in NIH research awards. *PLoS One* .2018;13(11) : 10.1371/journal.pone.0205929 e0205929 10.1371/journal.pone.0205929 30427864 PMC6235266

[6] : Experimentation by Industrial Selection. *Philosophy of Science* .2017;84(5) : 10.1086/694037 1008-1019 10.1086/694037

[7] : Epistemic diversity and industrial selection bias. *Synthese* .2023;201(5) : 10.1007/s11229-023-04158-7 10.1007/s11229-023-04158-7

[8] : On the very idea of pursuitworthiness. *Stud Hist Philos Sci* .2022;91: 10.1016/j.shpsa.2021.11.016 103-112 10.1016/j.shpsa.2021.11.016 34896786

[9] : Gender and racial/ethnic disparities in rates of publishing and inclusion in scientific-review processes. *Translational Issues in Psychological Science* .2021;7(4) : 10.1037/tps0000253 451-461 10.1037/tps0000253

[10] : How unpredictable is research impact? Evidence from the UK’s Research Excellence Framework. *Research Evaluation* .2023;32(2) : 10.1093/reseval/rvad019 273-285 10.1093/reseval/rvad019

# Reviewer report: Reviewer response for version 3

**DOI:** 10.5256/f1000research.162827.r277367

**Published:** 2024-05-24

**Authors:** Gerald Schweiger

**Affiliations:** 1 Graz University of Technology, Graz, Austria

Thank you for the opportunity to review this paper. I appreciate the effort that went into writing the paper and the thorough reviews. The author presents an interesting alternative idea for allocating scarce research funds. The ideas discussed in this paper are not only relevant to US funding agencies.

*My comments*

**(1)** I agree with Reviewer 3 regarding the term “ *science by consensus*”. At least it does not apply to all funding programs that projects are only funded if there is a consensus among the reviewers.

**(2)** „ *The peer review process that evaluates proposals has been claimed arbitrarily to be the best there is.*“ This is a strong claim. Indeed, some studies suggest that the grant review is arbitrary; other studies show at least very low to low agreement [1-3].

**(3) **„ *This consensus system, however, has never been evaluated scientifically against an alternative.*“ This is a strong claim. In recent years, work has been done to analyse alternative allocation systems (see e.g. [4-5]).

**(4)** „ *The success rate for National Institutes of Health (NIH) grants is currently 20% (NIH Report, 2022), and the “payline” for research grant applications by experienced principal investigators is 11% (NIAID Funding News, 2023).*“ It would be helpful to add a definition of „payline“.

**(5) **" *Some major breakthroughs in biomedical sciences have not been funded by NIH or NSF"*. I agree with Reviewer 3, I don't think this is a strong argument.

**(6) **" *Grant reviewers are competitors of applicants.“* This is a strong claim that should be backed up by literature. It does not seem to be a general argument (outside of NIH and NSF) since reviewers often have to be from abroad.

**(7)** “ *Criticism never ends.“ *This is a rather vague claim. Some literature exists on resubmissions (e.g., [6]).

**(8)** „ *Nonscientific, unpublished review criteria. Reviewers tend to use nonscientific criteria when making funding decisions. These include: [...] .*“ This is a strong claim that should be backed up by literature.

**(9) **„ *Precious scientist time is wasted on grant applications.*“  Instead of discussing a single study in detail, it might be better to summarize the findings of various studies. Our recent article discussed the economic costs of writing grant proposals [7]. Please note that this is not a suggestion to cite our paper but perhaps a source of relevant literature.

**(10) **„ *Scientists opting for this mode of funding would merely submit a letter of intent with a one-page broad outline of their research direction.*“ Writing a one-pager is not time-consuming, but if the content of this one-pager is not relevant to funding decisions, why not skip it and simplify the opt-in process?

**(11)** „ *It is theoretically possible, but extremely unlikely, that a scientist’s track record is exaggerated by the available data, [...].*“ I agree with Reviewer 3: Why is that so unlikely?

**(12)** The authors could suggest how potential risks could be analyzed in (smaller?) (if possibly controlled) experiments. A system change without such a prior analysis is somewhat unrealistic in my opinion.

**(13)** " *We contend that, since the ranking or evaluation system is without reviewer bias (there being no reviewers), it is orders of magnitude more reliable than the “science by consensus” system.*“ In this context, it is unclear to me what 'reliable' refers to.

**(14)** „ *Other alternatives to the peer review system.*“ This section needs to be improved by adequately describing the literature on these alternatives.

Is the topic of the opinion article discussed accurately in the context of the current literature?

Partly

Are arguments sufficiently supported by evidence from the published literature?

Partly

Are all factual statements correct and adequately supported by citations?

Partly

Are the conclusions drawn balanced and justified on the basis of the presented arguments?

Partly

Reviewer Expertise:

Science of Science; Intelligent Systems

I confirm that I have read this submission and believe that I have an appropriate level of expertise to confirm that it is of an acceptable scientific standard, however I have significant reservations, as outlined above.

## References

[1] : Peering at peer review revealed high degree of chance associated with funding of grant applications. *J Clin Epidemiol* .2006;59(8) : 10.1016/j.jclinepi.2005.12.007 842-8 10.1016/j.jclinepi.2005.12.007 16828678

[2] : Heterogeneity of inter-rater reliabilities of grant peer reviews and its determinants: a general estimating equations approach. *PLoS One* .2012;7(10) : 10.1371/journal.pone.0048509 e48509 10.1371/journal.pone.0048509 23119041 PMC3485362

[3] : When Zero May Not Be Zero: A Cautionary Note on the Use of Inter-Rater Reliability in Evaluating Grant Peer Review. *Journal of the Royal Statistical Society Series A: Statistics in Society* .2021;184(3) : 10.1111/rssa.12681 904-919 10.1111/rssa.12681

[4] : Peer review in funding-by-lottery: A systematic overview and expansion. *Research Evaluation* .2023;32(1) : 10.1093/reseval/rvac022 86-100 10.1093/reseval/rvac022

[5] Reference source

[6] : On the time spent preparing grant proposals: an observational study of Australian researchers. *BMJ Open* .2013;3(5) : 10.1136/bmjopen-2013-002800 10.1136/bmjopen-2013-002800 23793700 PMC3664356

[7] Reference source

# Reviewer report: Reviewer response for version 3

**DOI:** 10.5256/f1000research.162827.r277366

**Published:** 2024-05-16

**Authors:** Stijn Conix

**Affiliations:** 1 UCLouvain, Louvain-la-Neuve, Belgium

The author claims that the current system for distributing NIH and NSF funds has significant problems, including low success rates, potential biases, and inefficiencies. Despite claims of its effectiveness, this system has not been rigorously compared to alternatives. The author proposes to try out, on a large scale, an alternative system which provides long-term, stable funding based on scientific track records, without the need for detailed preliminary data or exhaustive peer review. This approach aims to reduce bureaucratic overhead and promote more equitable distribution of research funds.

 I should mention here that already before reading this paper I was highly critical of distributing funding by peer review of projects. Hence, I am not in the best position to be a very critical reviewer. It should also be no surprise then that I am reasonably sympathetic to the proposal of the author (as I would be of any system that drastically cuts the costs of distributing funding). However, I have a few problems with how the argument for this system is made in this paper:

 - Most importantly, the paper almost entirely ignores the substantial empirical literature on the topic. Much of this literature would support the author's claims, but some would not. I think the paper would be a lot stronger if it was embedded in this literature, and relied on the best evidence we have. This is currently not the case, and many empirical claims about the current system are made with very little empirical backing.

 - The paper does not give due credit to the current system and the people taking part in it. I am highly critical of this system, and am convinced that it should be changed and replaced by alternatives such as the one the author proposes, but I would be more charitable in describing the system, if only to enable productive debate with the proponents of the current system. The paper is written in a very opinionated and sometimes almost aggresive style, which will only hamper such debates.

 - I think it would be advisable to test a new system, like the one proposed here, before investing  30 billion dollars into it. For example, funders could set up small scale experiments and monitor outcomes. This way, multiple alternatives could be compared. It would be good if the author could explain why they don't choose such an approach.

 - The paper has very little discussion of other alternatives, such as various lotteries. I think it would be interesting to include such a discussion, as any alternative to the current system has to compete with these proposals to be tested.

 - The main shortcoming of the proposal, in my eyes, is that there is very little justification for the use of the criteria that the author proposes (h-index and other bibliometric measures). I agree that peer review is not reliable and lacks validity, but I have similar doubts about these alternative metrics as well. Indeed, peer review scores often are partially based on these metrics, so any reliability they have should partially show in peer review as well. The paper would be stronger if the author could give a clearer justification of their choice of criteria.

 In short, I am sympathetic to the author's proposal, but I think the argument could be made in a more rigorous, empirically substantiated and constructive manner.

 Small points:

 - Unclear what the 1 to 2% rate of the Gates foundation was based on. If it is pure speculation, it is better to omit it I think.

 - I'm not sure 'science by consensus' is the best possible name for this system, as consensus is neither necessary nor sufficient to get funding.

 - "Thus, the reputation of the current system is not based on systematic comparisons against other unbiased and more rational systems." While I agree, I don't think it is productive to claim the current system is less rational (and perhaps more biased) than alternatives. After all, you just claim that there has been no systematic comparison. Perhaps just omit 'unbiased and more rational'.

 - " Regrettably, study sections have had too much power over what projects should proceed and which ones should be scrapped. As we have stated before, “such ‘science by consensus’ is unhealthy for the unfettered and productive pursuit of biomedical science” (Düzgüneş, 1999)." It would be good to give reasons, rather than just state this claim. The one example you give is not a very strong reason.

 - "Furthermore, NIH has to process over 80,000 applications a year, utilizing over 25,000 reviewers (NIH Peer Review, 2019), an extremely wasteful system, possibly costing $1 billion/year, including the efforts of grant applicants and their post-doctoral fellows, graduate students and administrative assistants (Pagano, 2006)." Perhaps compare this to the budget that is being distributed and with estimates of the cost of review in other countries (I add a reference below).

 - "Some major breakthroughs in biomedical sciences have not been funded by NIH or NSF". While this is interesting, I'm not sure it is a strong reason. Scientific success is indeed hard to predict, but even if not all success can be predicted by peer review it might still be that overall it does a good job. To be clear: I don't think it does, but I also don't think peer review should be able to identify every potential noble prize winner in order to be a viable system.

 - " *Grant reviewers are competitors of applicants." *This is an empirical claim that is not trivially true: many reviewers are from other countries, and panel members have to follow conflict of interest rules (and perhaps cannot apply themselves?). It would be good to have some kind of substantiation of this problem, rather than making readers take your word for it.

 - "Thus, the NIH and NSF may not be funding discoveries, but merely funding “mopping up operations,” in the words of Thomas Kuhn (1962)". It is strange to cite Kuhn for this, as he did think that normal science is incredibly productive and essential to the success of science. There is much progress to be made within Kuhnian normal science, and I see no reason why NIH and NSF would not want to fund discoveries in normal science. If you really adhere to Kuhn's view on science, there are no reasons to hope that all science cosists in paradigm shifts.

 - "Grant applicants may re-apply after revising their proposal to respond to the written critique of the review panel. However, the panel may have new members at this later time and may then have entirely new criticisms. In essence, if the review panel does not want to fund an application, it will find ways not to fund the application, revealing the whims of the individual reviewers." The reasoning in this paragraph was not clear to me. It would also be good to discuss existing data on the success rate of resubmitted applications.

 - "Early career reviewers trained in a narrow area of science often think that valid science is what they are trained in. ..." The point that peer review is conservative is interesting, but does not seem related to the paragraph before that. Perhaps make this a separate point?

 - *Nonscientific, unpublished review criteria: *It is not clear to me what scientific review criteria would be, and why only scientific criteria are relevant. For example, potential long term societal relevance seems very important, but is not a scientific criterion. The discussion of interrater reliability is very brief, and only cites on of the very many studies on the subject. I recommend citing and reading the recent meta-analysis on the subject, and perhaps making this a separate point (see references).

 - "An example of this problem with NIH peer review is our inability to obtain grant funding since the mid-2000s for our research to develop gene therapy for oral cancer based on our initial discoveries ( Neves *et al.,* 2009), despite many applications." I think the paper would be stronger if the arguments relied less on single examples.

 - In a recent survey my co-authors and I did on QRPs in research funding, we had one question about this problem. It could be relevant to mention that result here. 

 - *The human and material cost of NIH peer review. *and *Precious scientist time is wasted on grant applications * could be combined, as they are both about the costs of the system (as is another paragraph higher up).

 - *NIH scientists do not compete for grant funding.* I do not see why a system could be good in some cases and not in others. In fact, I am convinced that we should use different distribution systems depending on different things we hope to achieve.

 - *Review panel scores do not predict success. *There is a lot of debate about this, and the phrasing here is too strong to reflect what the literature shows. There are some studies that show that review scores are somewhat predictive. I recommend including more references, and definitely some that disagree with your claim.

 - I might have miscounted, but I saw only 14 italicized problems.

 - Interventions to peer review: there is a substantial literature on potential interventions to peer review. As far as I know, none of these interventions are particularly successful, but it would be good to discuss this literature a bit.

 - A simple and rational alternative to peer review: I fail to see why this system is more rational than peer review (unless the 'rational' means 'cheaper'). That is, I see no reason to think that the criteria that the author proposes are more reliable in predicting success than peer review is. If I remember correctly there is a study that shows that bibliometric measures are better predictors of scientific success than peer review scores, but if this is the argument that the author is relying on, they should mention it (see refs)

 -   "It is theoretically possible, but extremely unlikely, that a scientist’s track record is exaggerated by the availalble data": Why is this unlikely? There is evidence that scientists 'game the metrics' by salami-slicing publications and boosting their track records as much as possible. The system proposed here would encourage such behaviour, and I don't see why that would be extremely unlikely.

 - "We contend that, since the ranking or evaluation system is without reviewer bias (there being no reviewers), it is orders of magnitude more reliable than the “science by consensus” system." I don't see why this would be the case. Even if there would be no reviewer bias, we'd need independent reasons to think that the new decision criteria are reliable.

Is the topic of the opinion article discussed accurately in the context of the current literature?

No

Are arguments sufficiently supported by evidence from the published literature?

No

Are all factual statements correct and adequately supported by citations?

Partly

Are the conclusions drawn balanced and justified on the basis of the presented arguments?

Partly

Reviewer Expertise:

Philosophy of science; science of science; science funding; philosophy of biology;

I confirm that I have read this submission and believe that I have an appropriate level of expertise to state that I do not consider it to be of an acceptable scientific standard, for reasons outlined above.

## References

[1] : Prior publication productivity, grant percentile ranking, and topic-normalized citation impact of NHLBI cardiovascular R01 grants. *Circ Res* .2014;115(7) : 10.1161/CIRCRESAHA.115.304766 617-24 10.1161/CIRCRESAHA.115.304766 25214575 PMC4163934

[2] : When Zero May Not Be Zero: A Cautionary Note on the Use of Inter-Rater Reliability in Evaluating Grant Peer Review. *Journal of the Royal Statistical Society Series A: Statistics in Society* .2021;184(3) : 10.1111/rssa.12681 904-919 10.1111/rssa.12681

[3] : Questionable research practices in competitive grant funding: A survey. *PLoS One* .2023;18(11) : 10.1371/journal.pone.0293310 e0293310 10.1371/journal.pone.0293310 37917785 PMC10621923

[4] : Research funding. Big names or big ideas: do peer-review panels select the best science proposals?. *Science* .2015;348(6233) : 10.1126/science.aaa0185 434-8 10.1126/science.aaa0185 25908820

[5] : Australia's grant system wastes time. *Nature* .2013;495(7441) : 10.1038/495314d 10.1038/495314d 23518554

# Reviewer report: Reviewer response for version 3

**DOI:** 10.5256/f1000research.162827.r249044

**Published:** 2024-03-21

**Authors:** Alejandra Recio-Saucedo

**Affiliations:** 1 National Institute for Health and Care Research (NIHR) Coordinating Centre, University of Southampton, Southampton, UK

I acknowledge the author's thorough response to my previous review.

 The paper undeniably proposes an interesting alternative to the current funding system. However, some key aspects of this alternative require further clarification before considering it fully viable. I’d like to note the following points that could be elaborated in future revisions of the alternative proposed:

**Early Career Researchers (ECRs):** A publication-based ranking system might disadvantage ECRs due to their limited publication history. How would the new funding model address this challenge?

**Unintended consequences:** Researchers with career breaks or those who don't follow traditional research paths may be disadvantaged. Gaps in publication track record or limited number of publications are common in these groups. What measures would be put in place to counter these potential biases in the alternative model?

**Research culture:** Initiatives like DORA promote assessment of broader research contributions which look beyond publications (e.g., teaching, mentoring, data sharing). How does this alternative system acknowledge and support non-publication outputs of technicians, data scientists and others who make important contributions to research through other types of outputs (e.g., research software, datasets, economic models)?

**Alternatives to peer review:** The alternatives described in the literature aim to move beyond *traditional* peer review (this open peer review is part of these efforts). The collective knowledge of the scientific community plays a role in the research ecosystem and it’s too valuable to not utilize. It is not necessarily ‘grasping at straws to save peer review’. It is merely learning from current systems to try to design the best alternative to fund research whilst acknowledging the limitations of human enterprise.

**Reviewer effort:** The proposed model would require uncompensated review work from international panels, who are ineligible to apply for funds under the alternative model. In addition, the mention of revising ranking criteria (potentially relaxing them) suggests the need for reviewer meetings across time zones to make funding decisions. The design of funding panels in this way raises concerns about volunteer burden. Furthermore, reviewer contributions, whether they involve two hours or two days must be recognized, and I do not necessarily agree with qualifying this work as minuscule.

**Training: **Familiarity with metrics like d-index and h-index might require training for reviewers.

**Evidence-based approach:** Addressing the limitations of peer review is commendable. However, determining the best alternative requires objective evidence comparing outcomes such as effort, fairness, cost and outputs/impacts of the research funded. This would guide the design of a system that truly benefits the research community, fosters scientific progress and supports delivery of societal benefits from research. An RCT instead of a 5-10 year evaluation as proposed might provide evidence in a shorter period of time.

Is the topic of the opinion article discussed accurately in the context of the current literature?

Partly

Are arguments sufficiently supported by evidence from the published literature?

Partly

Are all factual statements correct and adequately supported by citations?

Yes

Are the conclusions drawn balanced and justified on the basis of the presented arguments?

Partly

Reviewer Expertise:

I am Senior Research Fellow at the National Institute for Health and Care Research within the Research on Research group and have conducted research on peer review.

I confirm that I have read this submission and believe that I have an appropriate level of expertise to confirm that it is of an acceptable scientific standard.

# Reviewer report: Reviewer response for version 2

**DOI:** 10.5256/f1000research.159084.r227840

**Published:** 2024-02-14

**Authors:** Alejandra Recio-Saucedo

**Affiliations:** 1 National Institute for Health and Care Research (NIHR) Coordinating Centre, University of Southampton, Southampton, UK

The author reviewed the manuscript based on some of the recommendations made in the original submission and that is appreciated. However, the questions raised in the first review were answered only partially and concerns about the alternative proposed remain.

 The section on unintended consequences seems to indicate that the misidentification of track records for some applicants would be, albeit unlikely according to the author, inevitable. This because instead of offering a mechanism to avoid such consequence, the author offers a comparison of this consequence to what already occurs with the current funding allocation system. In addition, the solution proposed for the other unintended consequence discussed, this is, applicants not making the cut off of scientific measures to establish a track record, is for applicants to resort to the current funding system, which the author strongly criticises. The purpose of identifying unintended consequences should be avoidance, otherwise, why change? Since it is not possible to predict the incidence of these unintended consequences, it is necessary to design solutions for the alternative proposed and not operate on the premise that, because it is unlikely to happen, then there is no reason to plan for a way to address the issue should it arise.

 The alternative proposed relies heavily on researchers’ track record. The impact that the alternative has on early career researchers and other groups that have been identified to be less likely to receive funds for research (and as a result have less publications which lead to the other criteria proposed by the author, e.g., citations, h-index) has not been addressed in the alternative proposed. Establishing the criteria without identifying individual applicants does not solve the issue that some groups have been historically disadvantaged and are not going to meet the cut off criteria being suggested.

 The lottery mechanisms for funding allocation which have been tried or implemented still make use of peer review to identify applications that meet the criteria of a funding call. Random allocation or lottery mechanisms address other issues related to funding decisions, but do not remove the peer review process entirely.

 I agree with the author that systems to fund research must be revised constantly to ensure transparency, efficiency and fairness in how decisions are made, and that there are opportunities to improve the current funding systems. However, the alternative proposed does not clarify the potential for research waste from duplication or poor scientific productivity.

 A study to compare the current funding system with alternatives (e.g., sandpits, random allocation, distribution of research funds based on track record, etc.) would be highly valuable.

Is the topic of the opinion article discussed accurately in the context of the current literature?

Partly

Are arguments sufficiently supported by evidence from the published literature?

Partly

Are all factual statements correct and adequately supported by citations?

Yes

Are the conclusions drawn balanced and justified on the basis of the presented arguments?

Partly

Reviewer Expertise:

I am Senior Research Fellow at the National Institute for Health and Care Research within the Research on Research group and have conducted research on peer review.

I confirm that I have read this submission and believe that I have an appropriate level of expertise to confirm that it is of an acceptable scientific standard, however I have significant reservations, as outlined above.

# Reviewer report: Reviewer response for version 2

**DOI:** 10.5256/f1000research.159084.r227841

**Published:** 2023-12-27

**Authors:** Ferric Fang

**Affiliations:** 1 Department of Laboratory Medicine and Pathology, University of Washington, Seattle, WA, USA

In this revised manuscript, the author has strengthened his critique of the peer review systems used by the NIH and NSF to award research grants.  However, he has failed to address a major flaw in his alternative funding scheme to offer ‘continuous and stable funding’ of $400,000 per year in direct costs to scientists ‘with a track record of stable publications’ and $150,000 per year to young investigators.

 As mentioned in my earlier review, a 2006 NIH analysis found that measures of citation productivity peaked at mean funding levels of $750,000 per year.  Thus, funding labs at a fixed level of $400,000 per year would reduce productivity for many labs, and this is not even accounting for the substantial effect of inflation since that time. The author criticizes this analysis because he argues that productivity should be measured relative to funding levels, but the fact remains that absolute productivity for many individual labs would decline under his scheme.  Moreover, there is considerable variation in productivity among laboratories, and the impact of funding restriction would be greatest for the most highly productive labs.  A study of more than 15 million researchers from 1996-2011 found that fewer than 1% of labs published at least one article per year throughout the study period, yet this small fraction accounted for 41.7% of all papers and 87.1% of all papers with more than 1,000 citations Ioannidis et al (2014 ^1^).  By providing a minimal funding level for all labs, the author’s alternative scheme would potentially cripple the most productive labs.  The author may argue that labs desiring higher levels of funding could opt out of the alternative scheme, but intensive competition among the most highly productive labs for a reduced portion of the funding pie would only intensify the unhealthy level of competition that we see today.

 The author cites the HHMI investigator program as an example of a successful alternative system that leads to higher levels of breakthrough intervention.  However, he fails to note that HHMI investigators currently receive more than $1 million in direct support each year and represent an elite group of scientists with established high levels of productivity.

 Lastly, I would point out that $150,000 per year would be woefully inadequate support for young investigators and place them at a significant disadvantage compared to more experienced investigators receiving $400,000 per year.  This is reminiscent of the NIH R29 FIRST award program initiated in 1986 to provide modest levels of funding to early-stage investigators.  This program was discontinued in 1997 when it was recognized that R29 recipients were less successful in obtaining subsequent funding compared to those who initially received a standard R01 research grant.

 In summary, I find the critique of grant peer review to be cogent but the proposed solution to be extremely problematic.

Is the topic of the opinion article discussed accurately in the context of the current literature?

Yes

Are arguments sufficiently supported by evidence from the published literature?

Yes

Are all factual statements correct and adequately supported by citations?

Yes

Are the conclusions drawn balanced and justified on the basis of the presented arguments?

Partly

Reviewer Expertise:

microbiology, molecular biology

I confirm that I have read this submission and believe that I have an appropriate level of expertise to confirm that it is of an acceptable scientific standard, however I have significant reservations, as outlined above.

## References

[1] : Estimates of the continuously publishing core in the scientific workforce. *PLoS One* .2014;9(7) : 10.1371/journal.pone.0101698 e101698 10.1371/journal.pone.0101698 25007173 PMC4090124

# Reviewer report: Reviewer response for version 1

**DOI:** 10.5256/f1000research.136253.r200648

**Published:** 2023-09-22

**Authors:** Alejandra Recio-Saucedo

**Affiliations:** 1 National Institute for Health and Care Research (NIHR) Coordinating Centre, University of Southampton, Southampton, UK

The author presents an interesting and important reflection on a debate that has been going on for quite some time: Is peer review the best mechanism ( *e.g.,* in terms of efficiency, fairness, ability to detect ground-breaking ideas, resistance to bias) to make research funding decisions? Given the worldwide use of this mechanism for a number of activities within the research landscape (research funding, academic promotions, publications), peer review has been extensively studied many times with the aim of finding alternative mechanisms to fund research, some of them having been more or less successful than others.

 In the article the author describes an alternative mechanism to distribute funds which would be relevant to US funding agencies. The potential impact of an efficient alternative to the use of peer review in funding decisions to the research practice is significant.

 Some of the key points in the article indicate:

- The author summarises peer review in research funding as ‘cumbersome, expensive, and counterproductive’
- The author presents 15 issues with the current peer review system (science by consensus), including: missing major scientific breakthroughs, unfair/unbiased process driven by competition from researchers working in fields similar/the same to that of the applicant, requirements for preliminary data, lack of accountability for reviewers, poor understanding of the value of trans-disciplinary proposals, poor reviewer agreement, lack of long-term funding, and others.
- The author proposes the alternative to be implemented with a view to evaluate its effect compared to the current peer review system in 5-10 years, also raising a challenge to senior management of major US research funders and leaders.

**Overall strengths**

 The article is well structured, interesting, and contributes to the literature on how to increase research efficiencies, decision-making transparency, and impact, which keeps large funding organisations across the world preoccupied and involved in constant efforts for improvement.

 Reading this led me to think about how I am indeed putting peer review into practice by writing my reflection on this article. At the same time I couldn’t avoid thinking that for all its challenges, peer review is a mechanism that encourages conversation, debate, and advances in scholarship, and how open peer review offers avenues to address some of the challenges associated with peer review discussed by the author.

**Areas for improvement**

 The literature used to illustrate some of the research in peer review is not particularly the most recent. Comprehensive analysis of alternatives to peer review, which covers issues associated to peer review at all stages of funding processes, are available (see for example Peter Kolarz, Anete Vingre, Aaron Vinnik, Antonio Neto, Carlos Vergara, Claudia Obando, Rodriguez, Kalle Nielsen, Laura Sutinen, Review of peer review. Online report. June 2023, https://www.ukri.org/wp-content/uploads/2023/07/UKRI-060723-Review-of-peer-review-Final-report-revs-v2.pdf). Alternatives that substitute peer review at the funding decision stage have been published ( *e.g.,* the use of random allocation mechanisms to allocate research funds: Avin S: Mavericks and lotteries. Stud Hist Philos Sci. 2019; 76: 13-23) as well as researchers’ views of some of these approaches (see for example:  Liu M, Choy V, Clarke P, Barnett A, *et al.*: The acceptability of using a lottery to allocate research funding: a survey of applicants.Res Integr Peer Rev. 2020; 5: 3).

 The description of the alternative is summarised since it refers to previous publications, but the summary misses the key challenges and unintended consequences of implementing the proposed alternative. Identification of unintended consequences will allow for the development of relevant indicators that would generate data/evidence to evaluate the efficiency of the alternative in comparison to the traditional use of peer review. In addition, the alternative proposed  does not seem to offer a new way of allocation research funds, as it includes the use of peer review in the way that is currently used ( *i.e.,* a panel of peers selects applications based on given criteria).

 I appreciate that the author is not bringing in the positives of peer review to this article and that this strategy adds emphasis to the argument presented, however the narrative highlights the inefficiencies and issues surrounding peer review without acknowledging that no system is likely to be a one-size-fits-all solution to all the challenges surrounding allocation of research funds. Recognising unintended consequences of alternatives is key and recognising the pros of peer review would actually support the alternative proposed (since it utilises peer review).

 In terms of the alternative proposed, there are some points that would benefit from clarification:

 The author writes: “Review panel scores do not predict success. An analysis of 102,740 funded grants has shown that percentile scores generated by NIH review panels for the applications are poor predictors of publication and citation productivity (Fang *et. al.,* 2016). Thus, the meticulous scoring process is essentially useless.”

 Given the broad range of impacts that result from research, and the efforts on responsible use of metrics that are addressing known issues with research culture, the conclusion that a scoring system is not useful based on its ability to predict publication and citation productivity is to some extent reductive. I understand that the author is presenting more issues around peer review, but the emphasis on publication and citation is running counter intuitively to how research culture is evolving.

 The proposed alternative reads: “They [applicants] could be chosen based on criteria including publications, citations and potential impact of their research field, by an international group of both established and young scientists who are not in a position to receive funding from NIH or NSF and are thus not competitors”

 The alternative offered here is a variation of peer review, but does not offer a different method to assess funding applications that decreases potential bias in decisions or increase inter-rater agreement of scorers. As written earlier, some aspects of the alternative are not different from the current way of selecting applications based on given criteria. The author suggests that funding decisions be made by “established and young scientists who are not in a position to receive funding”. Finding established and young scientists who cannot receive funding by the selected organisations to conduct the review and/or make funding decisions decreases the pool of potential reviewers. Thus the workload on eligible individuals can become unmanageable very rapidly. Another aspect is training those young scientists in assessing applications for awards/grants that they do not know in depth. There seems to be underlying risks to the proposed alternative that run the risk of affecting Early Career Researchers (ECRs) disproportionately. In addition, the author suggests that funding panels consider potential impact of their research field. How can potential impact be objectively assessed by reviewers/funding panels? How would criteria that takes into account publications and citations be supportive of ECRs whose list of publications would not be competitive compared to senior researchers? Are ECRs inadvertently ineligible to the alternative?

 “Thus, at a total cost of $28.6 billion the NIH could fund 80,000 such grants, with minuscule expenses for scientific review.”

 If the process to identify reviewers and go through the peer review process for the other half of the NIHR extramural funds remain in place, how will costs decrease to the point that they become negligible?

 Overall, this is a good paper that will benefit from including additional information on key challenges of a large-scale implementation of an alternative to the *de facto* mechanism used in the distribution/allocation of research funds, which currently also regulates all research and scientific activity (from funding to publication; recruitment and career progression).

Is the topic of the opinion article discussed accurately in the context of the current literature?

Partly

Are arguments sufficiently supported by evidence from the published literature?

Partly

Are all factual statements correct and adequately supported by citations?

Yes

Are the conclusions drawn balanced and justified on the basis of the presented arguments?

Partly

Reviewer Expertise:

I am Senior Research Fellow at the National Institute for Health and Care Research within the Research on Research group and have conducted research on peer review.

I confirm that I have read this submission and believe that I have an appropriate level of expertise to confirm that it is of an acceptable scientific standard, however I have significant reservations, as outlined above.

## References

[1] : Review of peer review. Online report. June 2023.2023; Reference source

[2] : From funding agencies to scientific agency: Collective allocation of science funding as an alternative to peer review. *EMBO Rep* .2014;15(2) : 10.1002/embr.201338068 131-3 10.1002/embr.201338068 24397931 PMC3989857

[3] : Recruitment of reviewers is becoming harder at some journals: a test of the influence of reviewer fatigue at six journals in ecology and evolution. *Research Integrity and Peer Review* .2017;2(1) : 10.1186/s41073-017-0027-x 10.1186/s41073-017-0027-x 29451533 PMC5803623

[4] : Peer review of health research funding proposals: A systematic map and systematic review of innovations for effectiveness and efficiency. *PLoS One* .2018;13(5) : 10.1371/journal.pone.0196914 e0196914 10.1371/journal.pone.0196914 29750807 PMC5947897

[5] : Reducing bureaucratic burden in research, innovation and higher education. *London* .2020; Reference source

[6] : Grant Peer Review: Improving Inter-Rater Reliability with Training. *PLoS One* .2015;10(6) : 10.1371/journal.pone.0130450 e0130450 10.1371/journal.pone.0130450 26075884 PMC4468126

[7] : Alternatives to peer review in research project funding: 2013 Update. *Santa Monica: RAND Corporation* .2013; Reference source

[8] : The Air We Breathe: A Critical Look at Practices and Alternatives in the Peer-Review Process. *Perspect Psychol Sci* .2009;4(1) : 10.1111/j.1745-6924.2009.01105.x 40-50 10.1111/j.1745-6924.2009.01105.x 26158832

[9] : The acceptability of using a lottery to allocate research funding: a survey of applicants. *Res Integr Peer Rev* .2020;5: 10.1186/s41073-019-0089-z 3 10.1186/s41073-019-0089-z 32025338 PMC6996170

[10] : Mavericks and lotteries. *Stud Hist Philos Sci* .2019;76: 10.1016/j.shpsa.2018.11.006 13-23 10.1016/j.shpsa.2018.11.006 31558205

# Response

**Published:** 2023-11-18

**Authors:** Nejat Düzgüneş

**Affiliations:** University of the Pacific, USA

Response to Dr. Recio-Saucedo

 We thank Dr. Recio-Saucedo for her insightful review of our paper.

 “At the same time I couldn’t avoid thinking that for all its challenges, peer review is a mechanism that encourages conversation, debate, and advances in scholarship, and how open peer review offers avenues to address some of the challenges associated with peer review discussed by the author.”

 We agree that “ *open* peer review” has its advantages in encouraging debate on peer review and on advances in research. When individuals are willing to be identified in *open* peer review, they are less likely to be harshly critical of a scientific proposal. When “peer review” is anonymous, and it does not present published evidence to support the “review”, it can become irresponsible and vicious.

 “The literature used to illustrate some of the research in peer review is not particularly the most recent. Comprehensive analysis of alternatives to peer review, which covers issues associated to peer review at all stages of funding processes, are available (see for example Peter Kolarz…June 2023,…”

 We hope it is appreciated that our original article was submitted in August 2022, about 10 months earlier than the analysis mentioned by the Reviewer. Nevertheless, in the revised article, we cite and discuss this paper as well as some of the other publications the Reviewer has suggested. All the revisions in the manuscript are indicated with Track Changes.

 “The description of the alternative is summarised since it refers to previous publications, but the summary misses the key challenges and unintended consequences of implementing the proposed alternative. Identification of unintended consequences will allow for the development of relevant indicators that would generate data/evidence to evaluate the efficiency of the alternative in comparison to the traditional use of peer review.”

 The reviewer refers to “the key challenges and unintended consequences of implementing the proposed alternative;” but does not give any examples. Nevertheless, this is an important point. As an unintended consequence, we may think of cases where the track records of a few awardees were misidentified, and they do not generate any significant publications during the 10 years of funding. We would argue, however, that the loss of scientific progress in these circumstances should be compared to the loss of productivity by a laboratory when it cannot renew a 3-year grant and all the trained personnel have to leave the laboratory.

 By contrast, we could envision a scientist who opted for the novel funding mechanism, but did not make the cutoff of scientific measures used to establish the track record. In this case, the scientist always has the option to apply for the competitive grants. This is the situation with the current peer review system in which about 80% of biomedical scientists find themselves. The frequency of such incidences could be quantified by the granting agency. Whether this analysis could be translated into the “efficiency of the alternate in comparison to the traditional use of peer review” begs the question, “is there *any *efficiency in the current peer review system?” Our answer would be “no.” We have added a section in the revised paper discussing the possible, but in our view minimal, unintended consequences of our alternative method of funding.

 “In addition, the alternative proposed  does not seem to offer a new way of allocation research funds, as it includes the use of peer review in the way that is currently used ( *i.e.,* a panel of peers selects applications based on given criteria).”

 We stated in our paper that the alternative method of funding scientists would be based on the track record of solid publications of the scientists, a system which we had first proposed in 1999 (Düzgünes ¸, 1999). In 2007, we added the mechanism to provide funding to young scientists starting their first independent positions in a university or a research institute (Düzgünes ¸, 2007). The reviewer interprets the work of the “international group of both established and young scientists who are not in a position to receive funding from NIH or NSF and are thus not competitors” as a form of peer review, possibly because of our use of the word “chosen.” We should have given more details of what determines a “track record of solid publications” *We did not intend this group of scientists to be involved in the choice of the scientists to receive funding*, *but merely to establish the track record criteria*. Examples of such criteria are citations, publications, the *h*-index and the *d*-index (Di Caro et al., 2012). The international panel of scientists would only be involved in establishing the overall weight of such criteria. We have modified the manuscript to clarify this aspect of our proposed system.

 “…however the narrative highlights the inefficiencies and issues surrounding peer review without acknowledging that no system is likely to be a one-size-fits-all solution to all the challenges surrounding allocation of research funds. Recognising unintended consequences of alternatives is key and recognising the pros of peer review would actually support the alternative proposed (since it utilises peer review).

 One of our main contentions here is that a system that has been acclaimed for decades as being the best method to allocate research funding, has not even been tested side-by-side against an alternative, unbiased, and more rational system. As we have delineated above, we have recognized the positive aspect of peer review by proposing that the unequivocally objective criteria for the new system would be discussed and established by an international panel of junior and established scientists. Further pros of peer review may be seen in the review of manuscripts submitted to journals, where errors in the research or in the analysis of data may be identified. Even so, the rejection of breakthrough articles by Karikó and Weissman on mRNA by reputable journals, as reported in the NY Times, is not a plus for the reputation of peer review even for journal articles.

 In terms of the alternative proposed, there are some points that would benefit from clarification:

 The author writes: “Review panel scores do not predict success. An analysis of 102,740 funded grants has shown that percentile scores generated by NIH review panels for the applications are poor predictors of publication and citation productivity (Fang *et. al.,* 2016). Thus, the meticulous scoring process is essentially useless.”

 Given the broad range of impacts that result from research, and the efforts on responsible use of metrics that are addressing known issues with research culture, the conclusion that a scoring system is not useful based on its ability to predict publication and citation productivity is to some extent reductive. I understand that the author is presenting more issues around peer review, but the emphasis on publication and citation is running counter intuitively to how research culture is evolving.

 We are merely stating the conclusions of Fang et al. (2016), which may be reductive, but they provide strong support for our contention that the supposedly objective scoring system of grant peer review (which, by the way, has not been tested against an alternative system) does not lead to success in terms of measurable quantities, such as publications and citations. The Reviewer has not explained what she means by the thesis that these metrics are “running counter intuitively to how research culture is evolving.” Nevertheless, we believe this issue is outside the scope of our article.

 The alternative offered here is a variation of peer review, but does not offer a different method to assess funding applications that decreases potential bias in decisions or increase inter-rater agreement of scorers. As written earlier, some aspects of the alternative are not different from the current way of selecting applications based on given criteria.

 In the revised manuscript, we have clarified the involvement of the international panel of scientists. They do not act as peer reviewers of any grant applicant or application. They merely set the objective criteria by which scientists applying for the new system are ranked, without knowing anything about the scientist, their field of research or the general topic of the research the scientist wishes to pursue.

 “Finding established and young scientists who cannot receive funding by the selected organisations to conduct the review and/or make funding decisions decreases the pool of potential reviewers.”

 The majority of scientists live in countries outside of the United States. Although some non-U.S. scientists receive funding from NIH or NSF, their number is extremely limited, and they would not be permitted to participate in the international panel.

 “Thus the workload on eligible individuals can become unmanageable very rapidly”

 As we have clarified above and in the revised manuscript, the work of the international panel is minuscule compared to that of the NIH study sections or NSF reviewers. The panel merely determines the criteria of acceptance, such as the percentage weight of the *d*-index, the number of citations, the number of publications, and other criteria (e.g. the *h*-index) they may consider to be useful. If such ranking is too rigorous and eliminates too many of the applicants (which would be calculated almost instantaneously), the panel would relax the criteria.

 “Another aspect is training those young scientists in assessing applications for awards/grants that they do not know in depth.”

 As we have explained above, no such training would be required of any scientist. Nevertheless, the members of the review panel would have to become familiar with the *d-*index and the *h*-index, and perhaps other criteria.

 “There seems to be underlying risks to the proposed alternative that run the risk of affecting Early Career Researchers (ECRs) disproportionately.”

 On the contrary, in the scheme we have proposed, early career researchers beyond their post-doctoral training would be given 5-year grants to establish their own researach programs, without spending most of their time applying for grants, and most likely failing. This would help young scientists in obtaining tenure and keeping their laboratories and trained personnel. By contrast, in the current system, the fierce competition with established scientists puts early career researchers at a perilous disadvantage, and force a majority of them to close their labs and move to other universities, research institutions or industry.

 In addition, the author suggests that funding panels consider potential impact of their research field. How can potential impact be objectively assessed by reviewers/funding panels? How would criteria that takes into account publications and citations be supportive of ECRs whose list of publications would not be competitive compared to senior researchers? Are ECRs inadvertently ineligible to the alternative?

 We agree that considering the potential impact of the research field would be difficult; thus, we have deleted this expectation of the international panel. Regarding the ECRs, we have addressed this concern in the previous point above. In contrast to the Reviewer’s contention, ECRs are *currently* at an enormous disadvantage, because they are expected to compete with all the established investigators. In the system we are proposing, ECRs have the opportunity to set up their independent research with the help of the 5-year grant they will be given when they start their first independent position. Depending on the NIH and NSF budgets, the period and the amount of funding to the ECRs could be increased from those we are proposing here.

 If the process to identify reviewers and go through the peer review process for the other half of the NIH extramural funds remain in place, how will costs decrease to the point that they become negligible?

 The system we are proposing here would enable investigators whose research needs are much greater than the $400,000 per year, to compete for funds just like they have been doing for decades. The advantage of the split system we are proposing is that these investigators would not have to also compete with the 80,000 other applicants. We proposed the split system of funding with the expectation that the two segments could be compared at the end of a 5- or 10-year period, in terms of research productivity, breakthroughs and patents (Düzgüneş, 1999). The burden of peer review in study sections at NIH would be lowered, because there would be 80,000 fewer applications.

# Reviewer report: Reviewer response for version 1

**DOI:** 10.5256/f1000research.136253.r149008

**Published:** 2022-09-12

**Authors:** Ferric Fang

**Affiliations:** 1 Department of Laboratory Medicine and Pathology, University of Washington, Seattle, WA, USA

This commentary is a critique of the approaches used by the U.S. National Institutes of Health and National Science Foundation to allocate research funding. The commentary lists what it describes as 15 “major problems with the peer review process.” In place of the current system, the commentary suggests awarding funds at fixed levels based on level of applicant experience, a one-page summary of research plans, and publication record. This would reduce administrative costs and substantially increase the number of funded researchers. Few of the issues with grant peer review or the solutions proposed in this commentary are new ^1^^,^^2^^,^^3^.  Nevertheless, the topic remains timely, as low success rates for grant applications persist.

 1. The 20% success rate for NIH grants is a misleading indicator of the current difficulty in obtaining research funding, as success rates count original and resubmitted grants that are received during the same fiscal year as a single submission and combine different types of grants that have variable success rates. A better indicator would be paylines, which are at 10-12% for R01 applications from established investigators at many institutes. Thus, the situation is even more dire than the commentary suggests.

 2. There is some redundancy in the list of “major problems,” which essentially boil down to: (a) peer review is biased and unreliable; (b) the current funding system discourages innovation and long-term projects; (c) too much time is wasted on seeking funding or peer review instead of on research. Another concern, which is not mentioned, is that insufficient levels of public funding for research and development can have a disproportionate impact on female and underrepresented minority researchers ^4^^,^^5^^,^^6^.

 3. The commentary fails to consider potential disadvantages of the proposed funding strategy. For example, dramatically lowering the bar for research funding might result in an increase in lower quality research, because many labs whose work was not previously found to be sufficiently meritorious to warrant support would now be funded. Some institutions might be tempted to expand the size of their faculties in order to exploit the system, which would increase pressure on the research budget. Half of the extramural budget would be retained for competitive applications involving projects that require larger budgets, but this would essentially intensify competition among larger laboratories, which include some of the most productive research groups. The $400,000 per year budget falls far short of the $750,000 direct funding level that was found to be associated with peak measures of productivity in a 2006 NIH study ^7^, and research costs have only risen since that time, suggesting that most labs would be underfunded under the new scheme.

 4. The comparison of recipients of support from the NIH and Howard Hughes Medical Institute is invalid, because HHMI investigators represent a more highly selected subset of scientists with demonstrated high levels of productivity.

 5. The proposed funding approach shares some features with the approach proposed by Pagano ^1^, which should be cited.

 6. A single paragraph suggests performing a 5- and 10-year formal study to compare the productivity resulting from the current and alternative funding approaches but fails to consider the many methodological challenges in performing such a study.

 7. The commentary repeatedly refers to “we,” but only a single author is listed.

 Although I share many of the frustrations expressed in this commentary, I have concerns about the practicality of the proposed solution.  Nevertheless, a dialogue on possible ways to address the inadequate current levels of research funding and the shortcomings of grant peer review should continue. This commentary may help to further that dialogue.

Is the topic of the opinion article discussed accurately in the context of the current literature?

Yes

Are arguments sufficiently supported by evidence from the published literature?

Yes

Are all factual statements correct and adequately supported by citations?

Yes

Are the conclusions drawn balanced and justified on the basis of the presented arguments?

Partly

Reviewer Expertise:

microbiology, molecular biology

I confirm that I have read this submission and believe that I have an appropriate level of expertise to confirm that it is of an acceptable scientific standard, however I have significant reservations, as outlined above.

## References

[1] : American Idol and NIH Grant Review. *Cell* .2006;126(4) : 10.1016/j.cell.2006.08.004 637-638 10.1016/j.cell.2006.08.004 16923379

[2] : More time for research: fund people not projects. *Nature* .2011;477(7366) : 10.1038/477529a 529-31 10.1038/477529a 21956312

[3] : Research Funding: the Case for a Modified Lottery. *mBio* .2016;7(2) : 10.1128/mBio.00422-16 10.1128/mBio.00422-16 PMC495952627073093

[4] : Race, ethnicity, and NIH research awards. *Science* .2011;333(6045) : 10.1126/science.1196783 1015-9 10.1126/science.1196783 21852498 PMC3412416

[5] : Sex differences in application, success, and funding rates for NIH extramural programs. *Acad Med* .2011;86(6) : 10.1097/ACM.0b013e31821836ff 759-67 10.1097/ACM.0b013e31821836ff 21512358 PMC3379556

[6] : Inequalities in the distribution of National Institutes of Health research project grant funding. *eLife* .2021;10: 10.7554/eLife.71712 10.7554/eLife.71712 PMC844853234477108

[7] : Study says middle sized labs do best. *Nature* .2010;468(7322) : 10.1038/468356a 356-357 10.1038/468356a 21085145

# Response

**Published:** 2023-11-18

**Authors:** Nejat Düzgüneş

**Affiliations:** University of the Pacific, USA

Response to Dr. Ferric Fang

 We thank Dr. Ferric Fang for his thorough review of our paper. We greatly appreciate his conclusion that “Nevertheless, a dialogue on possible ways to address the inadequate current levels of research funding and the shortcomings of grant peer review should continue. This commentary may help to further that dialogue.”

 Our response to the Reviewer’s individual comments are given below. The changes in the manuscript are indicated by Track Changes.

 1. The 20% success rate for NIH grants is a misleading indicator of the current difficulty in obtaining research funding, as success rates count original and resubmitted grants that are received during the same fiscal year as a single submission and combine different types of grants that have variable success rates. A better indicator would be paylines, which are at 10-12% for R01 applications from established investigators at many institutes. Thus, the situation is even more dire than the commentary suggests.

 We have modified the Introduction to indicate this important point.

 2. There is some redundancy in the list of “major problems,” which essentially boil down to: (a) peer review is biased and unreliable; (b) the current funding system discourages innovation and long-term projects; (c) too much time is wasted on seeking funding or peer review instead of on research. Another concern, which is not mentioned, is that insufficient levels of public funding for research and development can have a disproportionate impact on female and underrepresented minority researchers

 We agree with the reviewer’s summary of the major problems with peer review; but we would prefer to retain the detailed exposé of peer review in our article. Regarding the disproportionate impact on female and minority resesarchers, we believe that our proposed system of grant allocation will alleviate the problem of recognition of the names of applicants by review panelists and the consequent bias against female and minority scientists. We have added a statement to this effect in the revised manuscript.

 3. The commentary fails to consider potential disadvantages of the proposed funding strategy. For example, dramatically lowering the bar for research funding might result in an increase in lower quality research, because many labs whose work was not previously found to be sufficiently meritorious to warrant support would now be funded. Some institutions might be tempted to expand the size of their faculties in order to exploit the system, which would increase pressure on the research budget. Half of the extramural budget would be retained for competitive applications involving projects that require larger budgets, but this would essentially intensify competition among larger laboratories, which include some of the most productive research groups. The $400,000 per year budget falls far short of the $750,000 direct funding level that was found to be associated with peak measures of productivity in a 2006 NIH study ^7^ , and research costs have only risen since that time, suggesting that most labs would be underfunded under the new scheme.

 We have added a section in the manuscript that discusses the unintended consequences of the proposed funding strategy, as also requested by Reviewer 2. Lowering the bar for research funding is highly unlikely to result in lower quality research, considering that a project that, for example, scores 10 points lower than the score of the funded grants is not expected to be of that much lower scientific quality. In support of this contention is the current inclination of some funding agencies to use a lottery to fund the grant applications in the top tiers.

 The reviewer in essence admits that large laboratories obtain the large grants, leaving individual, creative scientists (who may not be good academic politicians), without funds. In the revised manuscript, we have cited the recent case of Nobel Laureate Dr. Katalin Karikó, who could not obtain funding during the period of her groundbreaking work on modified mRNA. The alternative system we are proposing would enable scientists like Dr. Karikó to do their work without having to become a junior member of a large lab.

 The study reported in Wadman (2010), cited by the reviewer, regarding the high productivity of a lab with funding of $750,000 in direct costs is very misleading. The data presented in the article indicate that the productivity index of a lab with $400,000 is 6, whereas that of a lab with $750,000 is about 7.5. When expressed in terms of productivity per 1000 dollars, however, the $400,000 lab has a productivity index/1000 dollars of 0.015, compared to 0.01 for the lab with higher funding. This is 50% higher productivity per dollar!

 4. The comparison of recipients of support from the NIH and Howard Hughes Medical Institute is invalid, because HHMI investigators represent a more highly selected subset of scientists with demonstrated high levels of productivity.

 We cite this example only to illustrate the advantages of long-term funding, which allows for long-term and risky projects that may not produce results in a 3-year cycle. It is essential for policy-makers to implement such alternatives that will potentially produce results with high impact on understanding and curing diseases.

 5. The proposed funding approach shares some features with the approach proposed by Pagano ^1^ , which should be cited.

 We have cited Pagano (2006) regarding the impracticality of describing experiments to be done in 5 years, and the estimated total cost of writing and reviewing grant applications.

 6. A single paragraph suggests performing a 5- and 10-year formal study to compare the productivity resulting from the current and alternative funding approaches but fails to consider the many methodological challenges in performing such a study.

 The reviewer mentions the many methodological challenges in comparing the current and alternative funding approaches, but does not provide any examples. We propose that the international panel that sets the criteria for funding in the alternative system would use similar criteria, but weighted differently to also include patents, significant discoveries with high impact and developed therapeutics. We have also indicated provisions for center grants focusing on a particular disease.

 7. The commentary repeatedly refers to “we,” but only a single author is listed.

 The use of “we” is common practice in scientific writing. We have also cleared this with the Editorial Team at F1000.
